# Firefighters’ Occupational Exposure in Preparation for Wildfire Season: Addressing Biological Impact

**DOI:** 10.3390/toxics12030201

**Published:** 2024-03-05

**Authors:** Filipa Esteves, Klara Slezakova, Joana Madureira, Josiana Vaz, Adília Fernandes, Simone Morais, Maria do Carmo Pereira, João Paulo Teixeira, Solange Costa

**Affiliations:** 1 Environmental Health Department, National Institute of Health, Rua Alexandre Herculano, n° 321, 4000-055 Porto, Portugal; filipa.esteves@insa.min-saude.pt (F.E.); joana.madureira@insa.min-saude.pt (J.M.); solange.costa@insa.min-saude.pt (S.C.); 2 EPIUnit-Instituto de Saúde Pública, Universidade do Porto, Rua das Taipas, n° 135, 4050-600 Porto, Portugal; 3 Laboratório para a Investigação Integrativa e Translacional em Saúde Populacional (ITR), Universidade do Porto, Rua das Taipas, n° 135, 4050-600 Porto, Portugal; 4 Department of Public Health and Forensic Sciences, Medical School, Faculty of Medicine, University of Porto, Rua Doutor Plácido da Costa, 4200-450 Porto, Portugal; 5 LEPABE-ALiCE, Faculdade de Engenharia da Universidade do Porto, Rua Dr. Roberto Frias, 4200-465 Porto, Portugal; slezakok@fe.up.pt (K.S.); mcsp@fe.up.pt (M.d.C.P.); 6 CIMO, Instituto Politécnico de Bragança, Campus Santa Apolónia, 5300-253 Bragança, Portugal; josiana@ipb.pt; 7 SusTEC, Instituto Politécnico de Bragança, Campus de Santa Apolónia, 5300-253 Bragança, Portugal; 8 UICISA: E, Politécnico de Bragança, Campus de Santa Apolónia, 5300-253 Bragança, Portugal; adilia@ipb.pt; 9 REQUIMTE/LAQV-Instituto Superior de Engenharia, Instituto Politécnico do Porto, Dr. António Bernardino de Almeida 431, 4249-015 Porto, Portugal; sbm@isep.ipp.pt

**Keywords:** firefighters, non-invasive samples, biomonitoring, buccal micronucleus assay, particulate matter, inhaled dose

## Abstract

The characterization of wildland firefighters’ occupational exposure must consider different exposures, including those at the fire station. The present study aimed to characterize the occupational exposure of 172 Northern Portuguese wildland firefighters in fire stations during the pre-wildfire season of 2021. The biological impact of estimated inhaled doses of PM_10_ and PM_2.5_ (indoor/outdoor) was accessed through a buccal micronucleus cytome (BMCyt) assay in exfoliated buccal cells of a subgroup of 80 firefighters. No significant association was found between estimated inhaled doses of PM_10_ and PM_2.5_ (mean 1.73 ± 0.43 µg kg^−1^ and 0.53 ± 0.21 µg kg^−1^, respectively) and biological endpoints. However, increased frequencies of cell death parameters were found among subjects of the Permanent Intervention Teams (full-time firefighters). The intake of nutritional supplements was associated with a significant decrease in micronucleus frequencies (i.e., DNA damage or chromosome breakage). In addition, our findings showed a significantly increased frequency of cell death endpoints (i.e., nuclear fragmentation) with coffee consumption, while daily consumption of vegetables significantly decreased it (i.e., nuclear shrinkage). Our results provide data on the occupational exposure of wildland firefighters while working in fire stations during the pre-wildfire season, providing the essential baseline for further studies throughout the wildfire season.

## 1. Introduction

Occupational exposure as a firefighter was recently classified as “carcinogenic to humans” (Group 1) by the International Agency for Research on Cancer (IARC) [1]. However, only a few studies shed light on the exposure-induced biological mechanisms that may lead to such adverse health effects [2]. Wildland firefighters are typically exposed to a wide range of air pollutants during their activities [3]. In addition to fire suppression, firefighters may be exposed to various hazardous agents at fire stations, including particulate matter (PM). The presence of PM in fire stations can be attributed to various sources, including contaminated gear, equipment, and vehicles and diesel exhaust fumes originating from vehicles, fire engines, and trucks. This exposure may be particularly higher when facilities do not have adequate ventilation systems [4]. It is known that exposure to PM may induce various biological effects, including inflammation, DNA damage, and genomic instability [5]. Several studies have reported an association between human adverse health effects and exposure to PM_10_ and PM_2.5_ [6,7]. In the general population, PM_10_ exposure has been associated with respiratory illnesses, particularly in vulnerable groups [8], whereas exposure to PM_2.5_ has been linked with respiratory (e.g., chronic obstructive pulmonary disease and asthma) [9], cardiovascular (e.g., myocardial infarction, stroke, and arrhythmias) outcomes [10] and cancer [2]. Notwithstanding, few studies have focused on the biological impact of occupational exposure to PM on firefighters’ health [2]. So far, limited research exists regarding the assessment of firefighters’ occupational exposure to PM at fire stations [11,12,13], and up to date, none have studied its impact on effect biomarkers. 

Human biomonitoring studies can provide critical information on the potential risks occupational exposures may pose to human health [14,15]. The use of effect biomarkers is of utmost importance to detect early events associated with disease-related outcomes [14,15,16]. Classic cytogenetic assays, such as the micronucleus (MN) test, have been extensively used in molecular epidemiological studies [17]. MN frequency is a biomarker of chromosomal damage, genome instability, and cancer risk [17]. MNi are extra-nuclear DNA-containing bodies morphologically similar to the main nucleus but smaller, formed during cell division from chromosome fragments or whole chromosomes lagging at anaphase. Traditionally analyzed in blood, MNi have been successfully assessed in recent years in other non-invasive biological matrices, e.g., urine, nasal, and buccal cells. The use of MN frequency in exfoliated buccal cells has become particularly interesting due to its minimally invasive character [18] and because it has been strongly correlated with MN frequency in lymphocytes, an early cancer risk biomarker [19,20].

Moreover, since humans are nasal–oral breathers, the mouth may be the first tissue in contact with gaseous compounds during inhalation [21], making MNi in buccal cells an attractive biomarker to assess in occupational settings where workers are exposed to these agents, allowing the identification of individuals with a higher risk of developing adverse health outcomes (e.g., cancer of the upper aerodigestive tract) [14]. Several authors have found statistically significant correlations between MNi in peripheral blood lymphocytes and MNi in buccal cells of occupationally exposed populations [19,22]. This strong link can be interpreted as an indication that MNi in exfoliated buccal cells are also a predictive marker of cancer [20]. In addition, the buccal micronucleus cytome (BMCyt) assay, besides chromosomal instability, allows the detection/evaluation of other cytome parameters, such as gene amplification (nuclear buds frequency), cytokinetic defects (binucleated cells frequency), proliferative potential (basal cells frequency), and cell death parameters (condensed chromatin, karyorrhexis and karyolitic and pycnotic cells) [23,24]. 

The concurrent evaluation of the exposure and effect endpoints among wildland firefighters in different settings (i.e., non-fire; fire suppression activities) and different time-points of a wildfire season (e.g., before the season and during wildfire season) are of utmost importance for the health risk characterization of this carcinogenic occupation.

The present study aims to characterize wildland firefighters’ occupational exposure at fire stations before the wildfire season. The biological impact of estimated inhaled doses (indoor/outdoor) of PM_10_ and PM_2.5_ in the buccal cells of a group of Northern Portuguese wildland firefighters was assessed. In addition, the influence of demographic-, occupational-, and lifestyle-related variables on MN frequency and other BMCyt endpoints was evaluated. To our knowledge, this is the first study accessing MN frequency in buccal epithelial cells among wildland firefighters, as well as the first relating this effect biomarker with firefighters’ occupational exposure to PM_10_ and PM_2.5_, before the wildfire season.

## 2. Materials and Methods

### 2.1. Study Population 

Wildland firefighters (*n* = 176) with at least one year of service were recruited from fourteen Portuguese fire stations in Portugal’s northern region. All individuals who agreed to participate in the study were fully informed about the procedures and objectives of the study. Each participant signed an informed consent prior to the study. The research was conducted in full accordance with the Declaration of Helsinki [25,26]. Ethical approval for this study was obtained by the University of Porto Ethics Committee (reference number 92/CEUP/2020).

After giving written consent, participants were asked to complete a comprehensive questionnaire to obtain data on demographic-, occupational-, and lifestyle-related variables. Afterwards, participants were requested to provide one sample of blood, buccal mucosa cells, and urine, which were coded and analyzed under blind conditions. Confidentiality and privacy were guaranteed for all participants. Participants with cancer history (*n* = 1), with insufficient buccal cells to perform the BMCyt assay (*n* = 2), or with a missing questionnaire (*n* = 1) were excluded from the present analysis, reaching a total of 172 individuals. The biological and environmental samples were performed concurrently over two weeks in late spring of 2021.

### 2.2. Particulate Matter Monitoring

PM_10_ and PM_2.5_ were measured in seven fire stations before the wildfire season began (i.e., during the pre-fire season) over the period of two weeks in late spring of 2021. In each fire station, indoor and outdoor monitoring of PM were conducted concurrently over 1–2 consecutive days. Indoor PM levels were assessed by stationary monitoring within various communal indoor spaces, including rest areas and living quarters, which were commonly utilized as primary indoor areas where firefighters await deployment into service; in each indoor space, PM monitoring was conducted for at least 8 consecutive hours. Outdoor PM was continuously monitored in outdoor open-area zones directly adjacent to the main buildings, where workers regularly performed various technical or operational activities (e.g., equipment maintenance and vehicle services.).

Indoor and outdoor real-time PM (PM_10_ and PM_2.5_) concentrations were continuously monitored by a laser photometer (DustTrak^TM^ DRX, model 8533, TSI Inc., Minnesota, USA; measuring range of 1–150 × 103 µg m^−3^; accuracy of reading ± 0.1% 1 µg m^−3^) and a Lighthouse Handheld particle counter (model 3016 IAQ; Lighthouse Worldwide Solutions, Fremont, CA, USA; aerodynamic diameter of 300 nm–10 µm), respectively; one 1 min data resolution was used.

All equipment was positioned and mounted on supports at approximately 1.5 ± 0.2 m above the floor or ground surface and at least 1.5 m from the walls or any obstacles in order to minimize the influence on particle dispersion [27,28]. All direct emission sources that might interfere with data acquisition indoors and outdoors (i.e., air conditioners, ventilation points, entrance exits, walls, equipment, etc.) were avoided; outdoor equipment was positioned in a manner to be sheltered from direct sunlight and precipitation. 

Before the sampling campaign, all equipment was calibrated at the manufacturers. In order to minimize the occurrences of sudden artifact jumps in particle concentrations [29], all particle counters were zeroed daily (using external zeroing modules). 

Finally, all relevant information for indoor (e.g., layout, dimensions, construction materials, ventilation, and heating) and outdoor characterizations (e.g., levels of urbanization, presence of possible emission sources., etc.) of fire stations were collected. Other data, including occupancies and workers’ activities, were recorded.

### 2.3. Inhalation Dose Assessment

PM inhalation doses were estimated for 80 firefighters from the seven fire stations where air monitoring evaluation took place, using data from the questionnaires on gender, age, body weight (kg), and time spent at the fire station per day. The calculation of PM inhalation doses was performed based on methods described elsewhere [30]; the following equation was used:

Dose (D) = (BR/BW) × C × t,

where D corresponds to the age-specific dose (µg kg^−1^); BR is the age-specific breathing rate (m^3^ min^−1^); BW is the individual body weight (kg); C is the concentration of the PM in the respective indoor or outdoor spaces (µg m^–3^) (Supplementary Table S1), and t represents the time of exposure (min/day) (i.e., time spent at the fire station/day), considering a permanence of 70% outdoors and 30% indoors (Table S1). Mean BR was obtained from the US Environmental Protection Agency (Supplementary Table S1) [31]. 

The schedules and associated activities were registered and analyzed daily. In general, the study participants’ daily activity patterns were similar.

### 2.4. BMCyt Assay

#### 2.4.1. Reagents

Sodium chloride (NaCl), sodium hydroxide (NaOH), ethylenediaminetetraacetic acid tetra (EDTA), hydrochloric acid (HCL), Schiff’s reagent, Fast green FCF for microscopy, and Entellan^TM^ were acquired from Merck KGaA (Darmstadt, Germany). Tris-hydrochloride (Tris-HCl) was obtained from AppliChem (Darmstadt, Germany). Ethanol absolute and acetic acid glacial were purchased from VWR Chemicals (Radnor, PA, USA).

#### 2.4.2. Collection of Exfoliated Buccal Mucosa

Prior to buccal cell collection, subjects were asked to rinse their mouths with tap water to remove unwanted debris. Buccal sampling was performed separately inside both cheeks, left and right, to eliminate any unknown bias caused by sampling only one cheek. A sample from each cheek (left and right) was collected for every subject using separate cytobrushes (Deltalab) and suspended in 10 mL of cell buffer (0.01 M Tris-HCl, 0.1 M EDTA, 0.02 M NaCl, pH7). After collection, all biological samples were transported in a cooler (4 °C) to the laboratory for further processing. Samples were washed twice at 501 rcf with fresh cell buffer; after the last wash, cells were resuspended in 5 mL of a fixative solution of ethanol: acetic acid (3:1, *v*/*v*) at −20 °C and stored until further analysis.

#### 2.4.3. BMCyt Assay

**Figure 1:**
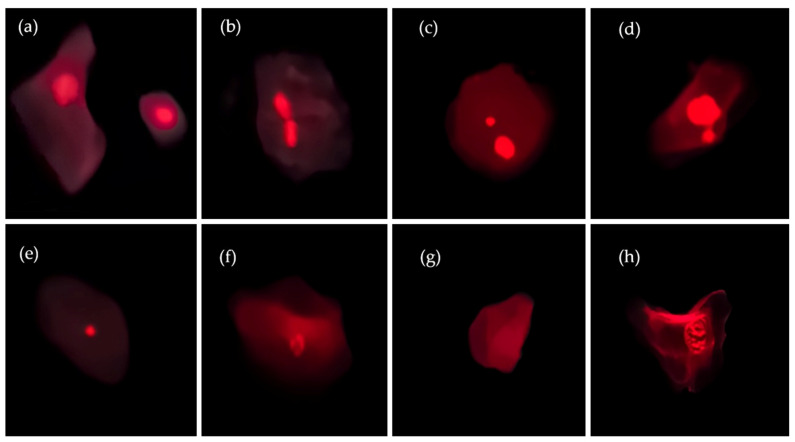
Photographic images of exfoliated buccal cells: (**a**) differentiated and basal cells; (**b**) binucleated cell; (**c**) differentiated cell with micronuclei; (**d**) NBUDs; (**e**) pycnotic cell; (**f**) karyorrhectic cell; (**g**) karyolitic cell; (**h**) differentiated cell with condensed chromatin.

BMCyt assay was performed according to the procedure described by Thomas et al. [23] and Costa et al. [22] with minor modifications. Briefly, fixed cells were centrifuged at 501 rcf for 10 min, and the pellet was dropped onto clear coded glass slides (three slides per cheek). Slides were left to dry horizontally overnight. Air-dried fixed slides were immersed in solutions containing 50% (*v*/*v*) and 20% (*v*/*v*) ethanol for 1 min each and later submerged in deionized water for 2 min. Next, slides were immersed in a 5M HCl solution for 30 min and washed in running tap water for 3 min. After that, slides were stained with Schiff’s reagent (Merck) at room temperature in the dark for 90 to 120 min. Slides were then counterstained for 5 sec in 0.2% (*w*/*v*) Fast Green (Merck) solution, dipped 3x in absolute ethanol, allowed to air dry, and mounted with Entellan^®^. The Feulgen-Fast green staining avoids false positive results (commonly found in non-specific DNA staining analysis, such as Giemsa) and consequently contributes to reliable and comparable results. Slides were scored blindly by a single trained operator on a Nikon Eclipse E400 light microscope under 400× magnification. Cells containing micronuclei were confirmed under fluorescence using a G-2A filter to eliminate false positives. Scoring was performed according to criteria proposed by previous studies [23,24]. The frequency of basal cells, binucleated cells (BN), cells undergoing death, such as cells with condensed chromatin, karyorrhectic cells, karyolitic cells, and pycnotic cells was determined in 1000 cells, whereas the genetic instability and gene amplification biomarkers (MNi and NBUDs) were scored in 2000 differentiated cells [23] per individual. In this study, we converted the MN frequency units to MN frequencies per 1000 cells (‰). Photographic images obtained during the present analysis, showing typical examples of cells scored in the BMCyt assay, are presented in Figure 1. 

### 2.5. Statistical Analysis

A descriptive analysis was performed both for quantitative and qualitative variables. The normality of continuous variables was determined using the Kolmogorov–Smirnov test, complemented with data dispersion graphs and outlying values analysis. *T*-test (for continuous variables) and chi-square test of independence (for categorical variables) were used for the comparison between groups (i.e., total group and subgroup from the seven fire stations where PM measurements were conducted). Body mass index (BMI) [weight (kg) × height (m)^−2^] and nutritional status (i.e., underweight, normal weight, overweight, and obese) were calculated and classified according to the World Health Organization [32]. Concerning smoking habits, subjects were classified as smokers, non-smokers, or ex-smokers. Ex-smokers were defined as those who had quit smoking at the time of the interview and had smoked at least 100 cigarettes during his/her lifetime [33]. Dependent variables (BMCyt assay outputs) departed from the normal distribution, so non-parametric tests were used. Differences were analyzed by the Man–Whitney U-test (two groups) and the Kruskal–Wallis test (more than two groups) with the Bonferroni adjustment. Spearman’s rank correlation test was used to determine any possible correlation between BMCyt assay parameters and other continuous variables. In addition, simple linear regression analyses were performed to investigate the possible influence of independent variables (e.g., demographic-, occupational-, and lifestyle-related) on the BMCyt assay outputs. Categorical variables were transformed into dummy variables. A significance level of 0.05 was considered. All statistical analyses were performed using IBM SPSS Statistics 26.0 for Windows (IBM Corp., Armonk, NY, USA).

## 3. Results

### 3.1. Population Characterization

Table 1 presents the general characteristics of the study participants. No significant differences were observed between the study population and the subgroup.

BMI values among males and females were 27.6 ± 3.9 kg m^−2^ and 26.4 ± 4.5 kg m^−2^, respectively. According to World Health Organization criteria [27], only 29% of firefighters (*n* = 47) had normal weight (range, 18.5–24.9 kg m^–2^), with the remaining 44% (*n* = 71) and 27% (*n* = 43) being either pre-obese (range, 25.0–29.9 kg m^–2^) or obese (≥30.0 kg/m^2^), respectively. Most firefighters (75%) declared having some physical activity. Nearly 90% of the firefighters reported being secondhand smokers (i.e., those exposed to environmental tobacco smoke, also called passive smokers) mostly at work and during leisure activities. Regarding dietary behaviors and practices, the majority of firefighters consumed vegetables daily, coffee, and smoked food between 2–3 times a week. 

Around 15.0% of the firefighters reported starting their training between the ages of 12 and 16 (i.e., as a junior or a cadet, respectively). However, the majority, 55.8%, started between 17 and 22 years old, while the rest (29.4%) started after that age. Besides fire combat, firefighters reported being involved in other activities, namely telephone operator (*n* = 17; 11.1%), driver (*n* = 53; 34.6%), pre-hospital emergencies (*n* = 47; 30.7%), rescue (*n* = 12; 7.8%), and command tasks (*n* = 12; 7.8%). Concerning the average time spent at the fire station during the off-season, a meaningful proportion of participants (49.1%) reported spending more than 10 h daily in their duties at the fire station, 37.1% within 8–9 h, and the remaining, less than 8 h (13.8%). Most firefighters were volunteers, whereas 30.1% (*n* = 46) reported to be full-time contract employees and members of the Permanent Intervention Team.

### 3.2. PM Inhaled Doses vs. Effect Biomarker 

Detailed results of indoor and outdoor PM_10_ and PM_2.5_ levels measured in the seven fire stations were described previously by Slezakova et al. [34]. Mean concentrations of indoor PM_10_ ranged between 8 and 15 µg m^–3^ (overall mean: 10 µg m^–3^), while mean indoor PM_2.5_ concentrations at each fire station ranged between 6 and 13 µg m^–3^ (overall mean: 8.5 µg m^–3^). Regarding the outdoor concentrations of PM_10_ and PM_2.5_ measured in the seven fire stations, means varied between 16 to 27 µg m^–3^ (overall mean: 21 µg m^–3^) and between 2 and 6 µg m^–3^ (overall mean: 5 µg m^–3^) for PM_10_ and PM_2.5_, respectively. 

Table 2 presents the descriptive statistics regarding the estimated inhaled doses of PM_10_ and PM_2.5_ with the respective indoor and outdoor contributions (expressed as %). 

A higher mean of inhaled doses of PM_10_ was observed when compared with PM_2.5_. The mean of estimated inhaled doses due to exposure to PM_10_ outdoors was four times higher than indoors. For PM_2.5_, the contributions from indoor and outdoor exposures were similar.

Simple linear regression analyses were performed to investigate the influence of the estimated PM_10_ and PM_2.5_ doses on the frequency of the effect biomarker endpoints. No significant statistical association was found. Table 3 shows the data obtained for the MN frequencies.

### 3.3. Influence of Demographic, Occupational, and Lifestyle Variables on Effect Biomarker Endpoints

In the present study, the frequency of MNi in buccal cells was not significantly influenced by age, gender, lifestyle (e.g., smoking habits), or years of service. However, firefighters using nutritional supplements in the last year showed a significantly lower frequency of MN than those who did not (mean ± S.E., 0.16 ± 0.07 vs. 0.40 ± 0.04; *p* = 0.04). Furthermore, firefighters of the Permanent Intervention Team showed a significantly increased frequency of cell death parameters (i.e., nuclear fragmentation) compared to others (mean ± S.E., 3.54 ± 2.91 vs. 1.32 ± 0.10; *p* = 0.01). A significantly higher frequency of this endpoint was also found in firefighters who reported drinking coffee (mean ± S.E., 2.97 ± 0.96 vs. 0.25 ± 1.12; *p* = 0.02). Moreover, higher frequencies of nuclear shrinkage (cell death parameter) were observed among individuals consuming smoked meat daily (*p* = 0.03); this endpoint was also higher in firefighters who reported not eating vegetables (*p* = 0.05). Female firefighters revealed significantly higher frequencies of binucleated cells than males (mean ± S.E., 0.52 ± 0.15 vs. 0.11 ± 0.03; *p* < 0.01). Pre-obese firefighters presented a slight increase in karyorrhectic cells compared to those with normal weight (mean ± S.E., 0.14 ± 0.05 vs. 0.00 ± 0.00; *p* = 0.04).

**Figure 2:**
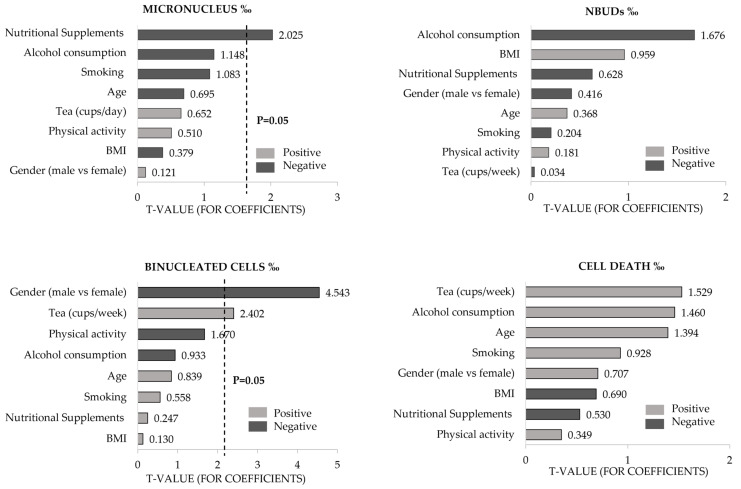
Simple regression analysis results considering the total sample (*n* = 172). Correlation between the frequency of each BMCyt endpoint (micronuclei, nuclear buds, binucleated cells, and sum of cell death endpoints) and selected predictor variables.

Simple regression analyses were applied to assess the extent of the effect of independent variables (e.g., demographic-, occupational-, and lifestyle-related variables) on the frequencies of micronucleus (MN), nuclear buds (NBUDs), binuclear cells, and cell death endpoints; the results of this analysis are displayed in the form of Pareto charts of t-values (Figure 2). The Pareto charts comprise a series of bars whose lengths indicate the impact of each predictable variable on the dependent variable. Due to the high number of tested variables, here, we only present important theoretical confounders that are typically used in these types of biomonitoring studies (i.e., age, gender, smoking habits, BMI, physical activity, and alcohol consumption) and variables that showed to be statistically significantly associated with some of the BMCyt parameters evaluated, namely the intake of nutritional supplements and tea (cups/week).

Some dietary habits influenced some BMCyt assay markers; the intake of nutritional supplements had a statistically significant negative contribution to micronuclei frequency (β = −0.24; *p* = 0.04), whereas ingestion of tea (cups/week) was a predictor of binucleated cells (β = 0.03; *p* = 0.02). Gender, i.e., female, was a predictor of the occurrence of binucleated cells (β = −0.41; *p* < 0.01).

## 4. Discussion

Wildland firefighters may be exposed to genotoxic compounds in different settings during their activities. When working at fire stations, firefighters can be exposed to diesel exhaust or other hazardous materials, such as PM and other known carcinogenic agents. Thus, any risk assessment of firefighters’ occupational exposure should include these activities. To our knowledge, no studies have yet estimated the inhaled doses of PM_10_ and PM_2.5_ (indoor/outdoor) at fire stations or even correlated them with a biomarker of effect. The frequency of MNi in buccal cells is particularly important as it has been proven to be a sensitive biomarker for detecting early biological effects of air pollutant exposure [35]. 

In this study, indoor PM_10_ and PM_2.5_ concentrations were below the levels set in the Portuguese Law No. 138-G/2021 (PM_10_ = 50 µg m^–3^ and PM_2.5_ = 25 µg m^–3^, respectively) that establishes the requirements for the assessment of indoor air quality in public buildings [36]. Regarding outdoor PM_10_ and PM_2.5_ levels, the observed concentrations fulfilled the recommendations proposed by the World Health Organization in 2021 (45 and 15 µg m^–3^, respectively) [37].

No association was found between inhaled doses (indoor/outdoor) of PM_10_ and PM_2.5_ and MN frequency, which could be related to the low PM levels.

The level of MN frequency found in our population was within the expected range of spontaneous events [0.30–1.70‰], according to Bonassi et al. [38]. Understanding the MN frequency distribution in a population is crucial [39], particularly if MN frequencies are to be used as biomarkers to evaluate the biological impact of workers’ exposure to hazardous agents [40].

Regarding the influence of variables on BMCyt endpoints, here, we observed that the number of years in service as a firefighter did not influence the dependent variables. Nevertheless, we found that subjects of the Permanent Intervention Teams had statistically significantly higher levels of karyolitic cells, a cell death parameter, compared to other colleagues. These teams comprise full-time contracted firefighters who may perform different tasks throughout the year, including firefighting, rescue, medical emergencies, vehicle maintenance, support to natural disasters, and other emergency response operations. The level of commitment, the extent of occupational exposure to harmful substances, and the stressful nature of their work may justify the difference found.

Aside from occupational exposure, there are endogenous (e.g., age and gender) and exogenous factors (e.g., smoking, drinking habits, and diet) that must be taken into consideration in any human biomonitoring study [19,41].

Some studies have shown that age may influence MN frequencies in buccal cells [42]; however, no association was found in the present study. Similar to our results, Haveric et al. [43] accessed the cytogenetic levels in exfoliated buccal cells in young individuals (aged 20–37) and found no association. In our study, no statistically significant differences were observed in MN frequency between males and females, which aligns with other studies [44,45]. The HUMNXL project (Human MicroNucleus project; ‘XL’ referring to eXfoLiated cells) evaluated the impact of host factors, occupation, and lifestyle on the occurrence of MNi in exfoliated buccal cells and also found no effect of gender [38]. Notwithstanding, in this study, a significant increase in binucleated cells was found among female firefighters. Few studies have mentioned differences concerning occupational aspects between female and male firefighters. Perroni et al. [46] reported that due to physiological sex differences, female firefighters have a higher level of physical demands to perform expected firefighter tasks. In addition, a working group of IARC recently concluded that a lack of proper personal protective equipment (PPE) fitting is likely among females, which could result in more significant contamination and exposure [2]. However, to date, a lack of evidence exists concerning the effect of gender in BMCyt endpoints, and therefore, caution is warranted when interpreting the results obtained here; further studies using BMCyt endpoints are needed.

Very few studies have explored the influence of BMI on the frequency of MNi in buccal cells [47]. Idolo et al. (2018) assessed the association between the frequency of MN cells and BMI categories among a group of one hundred and twenty-two healthy Italian children aged 6–8 years and found that obesity was strongly associated with this biomarker (OR = 3.849; CI = 1.140–12.994). Similarly to our results, Ernst et al. [48] found no association between BMI and MN frequency in a group of male individuals (aged 33.6 ± 8.6). Yet, we found that overweighted firefighters had statistically increased frequencies of cell death endpoints (i.e., condensed chromatin) compared with those with normal weight. 

Despite the numerous genotoxic chemicals in tobacco smoke [49], scientific findings on smoking and MN frequency are controversial [45,50]. Bloching et al. [50] found a strong positive correlation between smoking and MN frequency in buccal cells of tumor patients and healthy controls. However, most studies found no association between MNi in buccal cells and smoking habits [45,51]. Furthermore, in a pooled re-analysis of 24 databases of the HUMN international collaborative project [52], the authors found no effect of smoking nor the number of daily cigarettes on MN frequency analyzed in lymphocytes [52]. In agreement, our results also demonstrated that smoking, as well as the number of cigarettes smoked and the duration of smoking in years, did not have a significant impact on the frequencies of the studies’ endpoints. A possible explanation raised by some authors is related to a possible adaptive response (<30 cigarettes per day) showing that the MN frequency in lymphocytes was not influenced by the number of cigarettes smoked per day among subjects occupationally exposed to genotoxic agents [52].

Alcohol intake is a risk factor often collected in biomonitoring studies and has previously been linked to MN frequency in lymphocytes [53]. However, in line with previous works [54,55,56,57,58], alcohol consumption was not significantly associated with the increase in any BMCyt parameter. It is important to highlight that our study sample did not include any heavy alcohol drinkers; most firefighters reported drinking alcohol moderately (one to two cups of wine/beer).

The influence of tea and coffee consumption on BMCyt endpoints was also evaluated. Neither tea nor coffee intake was a predictor of buccal MN frequencies. Nevertheless, other BMCyt parameters, such as binucleated cell frequency and cell death endpoints (i.e., nuclear fragmentation), were significantly higher with tea (cups/day) and coffee intake (yes vs. no), respectively. A possible explanation for these findings may be related to the consumption of hot beverages; in fact, IARC classified hot beverage consumption as probably carcinogenic to humans [59]. Ernst et al. [48] found a significant association between temperature (>60 °C) and the increase in BMCyt endpoint frequencies in a group of 73 individuals before and after consuming hot beverages, i.e., tea and coffee.

Firefighters who regularly eat vegetables revealed statistically significantly lower cell death levels than those who never eat vegetables. In line with our results, results published by the HUMNXL project [38] revealed that the consumption of vegetables had a protective role on exfoliated buccal cells (lower MN frequencies in daily consumers when compared with those that reported no consumption at all). Conversely, firefighters who reported daily consumption of smoked food showed statistically higher frequencies of this endpoint; smoked food (e.g., ham and sausages) is known to be a dietary source of several hazardous compounds [60].

In this study, the intake of nutritional supplements was associated with lower MN frequencies. This finding is consistent with earlier reports; for example, Fenech et al. [61] demonstrated that low intake of calcium, folate, nicotinic acid, vitamin E, retinol, and beta-carotene and high intake of pantothenic acid, biotin, and riboflavin increased genome instability. In addition, the results of a review carried out by Thomas et al. [62] suggested that supplementation with antioxidant vitamins may cause a decrease in MN frequency both in buccal cells and in lymphocytes. The association of either nutritional supplement and vegetable intake with BMCyt assay outputs may be explained by the higher concentration of antioxidants and other nutrients that play an important role in genome health maintenance [63].

### Strengths and Limitations

The combination of biological monitoring with questionnaires and environmental monitoring data allows a comprehensive characterization of wildland firefighters before the wildland season. To the best of our knowledge, no studies have yet analyzed the impact of estimated inhaled doses of PM_10_ and PM_2.5_ (outdoor/indoor) in MN frequency or explored the influence of potential confounder variables or risk factors. Moreover, this is the first study accessing MN frequency in buccal epithelial cells among wildland firefighters; so far, only a previous work comprising urban firefighters (*n* = 47) has performed it [64].

However, our findings should be interpreted in light of some limitations. Data were collected through self-reported questionnaires; thus, some possible biases may have affected the variables (underestimate or overestimate). It is important to note that this study was conducted in a post-COVID-19 deconfinement period (2021) with an overall lower particulate air pollution. Thus, the association between the respective pollutants and biological markers may have been underestimated. In addition, as this is a cross-sectional study, it is impossible to establish a temporal relationship between exposure and the outcome. Still, this work offers valuable insights for guiding future research and stands as a foundational study for prospective longitudinal comparisons (e.g., pre-fire vs. during a fire season).

## 5. Conclusions

Our findings contributed to a baseline characterization of firefighters’ occupational exposure in preparation for the wildfire season. Given the multitude of PM sources (e.g., exhaust fumes from operational vehicles and contaminated PPE/equipment/vehicles), a comprehensive understanding of respective exposure within fire stations becomes imperative. The inclusion of a sensible biological endpoint, such as the BMCyt assay, may help to estimate the health risks faced by firefighters. We found no significant correlation between estimated inhaled doses of PM_10_ and PM_2.5_ and biological endpoints, as assessed by the BMCyt assay, probably due to the environmental levels found below established threshold values. Exposure to PM_10_ and PM_2.5_ has been linked to various adverse health effects, including respiratory and cardiovascular problems, cancer, as well as premature death. However, it is also worth noting that even exposure to levels below these limits may still pose health risks, and cumulative exposure effects over the years cannot be excluded. Improving air quality within fire stations through measures such as enhanced ventilation systems and regular air quality monitoring is essential for fostering healthier work environments.

The incorporation of sensitive biological endpoints, such as the BMCyt assay, can provide valuable insights into the health risks faced by firefighters. Our study revealed that certain occupational groups, such as full-time firefighters (Permanent Intervention Teams), exhibited higher levels of cell death endpoints compared to the other firefighters. Interestingly, we found that those reporting healthy dietary habits, particularly regular vegetable consumption, showed lower levels of cell death endpoints. Maintaining a healthy lifestyle, including proper nutrition and exercise, is crucial for enhancing overall health and resilience to occupational demands among firefighters. Moreover, it is imperative to implement occupational health and safety procedures at both individual and organizational levels.

In summary, (bio)monitoring programs are a crucial tool to identify firefighters at high risk for developing adverse health outcomes (short- and long-term). Further studies analyzing the two main points of firefighters’ occupational exposure, either during resting or off-season as during the fire season, are warranted to comprehensively evaluate occupational exposures holistically and refine strategies for safeguarding firefighter health and well-being.

## Figures and Tables

**Table 1:** General characteristics of the study population (*n* = 172) and subgroup from the seven fire stations where air monitoring evaluation occurred (*n* = 80).

Study Participant Characteristics			Total (*n* = 172)		Subgroup (*n* = 80)
		mv *		mv *	
Sociodemographic					
	Gender, *n* (%)	-		-	
	Females		31 (18.0%)		12 (15.0%)
	Males		141 (82.0%)		68 (85.0%)
	Age (years) **	2	37.5 ± 10.9 (19.0–65.0)	-	37.6 ± 11.9 (19.0–65.0)
	BMI (kg m^−2^) **	11	27.4 ± 4.1 (18.5–41.3)	-	27.7 ± 4.0 (18.5–41.3)
Occupational exposure:					
	Years of service (as a firefighter) **	7	15.9 ± 10.3 (1.0–43.0)	3	16.2 ± 11.5 (1.0–43.0)
Environmental exposure:					
	Secondhand smoke exposure, *n* (%)	7	144 (87.3%)	1	69 (87.3)
	X-ray exposure last year, *n* (%)	7	68 (41.2%)	1	32 (40.5%)
Lifestyle characterization:					
Alcohol intake, *n* (%)		3	53 (31.4%)	-	26 (32.5%)
	Smoking habits	2		-	
	Non-smoker, *n* (%)		74 (43.5%)		37 (46.3%)
	Ex-smoker, *n* (%)		34 (20.0%)		25 (22.5%)
	Current smoker, *n* (%)		62 (36.5%)		25 (31.3%)
	Physical activity, *n* (%)	3	128 (75.7%)	-	60 (75.0%)
Diet					
	Vegetable intake	23		8	
	Never, *n* (%)		12 (8.1%)		8 (11.1%)
	Daily, *n* (%)		101 (67.8%)		50 (69.4%)
	Weekly, *n* (%)		36 (24.2%)		14 (19.4%)
	Coffee intake, *n* (%)	1	147 (86.0%)	-	71 (88.8%)
	Nº times/week **	57	23.6 ± 13.4 (3.0–70.0)	24	23.2 ± 12.3 (3.0–56.0)
	Tea intake, *n* (%)	1	70 (40.7%)	-	32 (40.0%)
	Nº times/week **		5.2 ± 5.7 (1.0–28.0)		5.8 ± 6.6 (1.0–28.0)
	Smoked food consumption, *n* (%)	1	129 (75.4%)	-	61 (76.3%)
	Nº times/week **		3.1 ± 4.5 (1.0–35.0)		2.3 ± 3.1 (1.0–14.0)
	Nutritional supplements intake, *n* (%)	4	19 (11.3%)	-	11 (13.8%)

* Missing values. ** Mean ± SD (standard deviation), (min-max).

**Table 2:** Estimated inhaled doses of PM_10_ and PM_2.5_ for 80 firefighters from seven fire stations.

		Mean ± SD	Min.–Max.	Median	P25–P75
Inhaled Dose PM_10_ (µg kg^−1^)		1.73 ± 0.43	0.85–2.70	1.68	1.45–2.02
	Contribution of Indoor (%)	18.12 ± 4.08	10.67–26.79	18.00	17.06–17.95
	Contribution of Outdoor (%)	81.87 ± 4.08	73.21–89.33	82.42	82.05–82.94
Inhaled Dose PM_2.5_ (µg kg^−1^)		0.53 ± 0.21	0.22–1.09	0.49	0.37–0.72
	Contribution of Indoor (%)	49.09 ± 9.09	26.22–57.69	54.00	44.74–53.94
	Contribution of Outdoor (%)	50.90 ± 9.09	42.31–73.78	46.39	46.05–55.26

SD: standard deviation; Min: minimum; Max: maximum; P25: percentile 25; P75: percentile 75.

**Table 3:** Influence of inhaled PM_10_ and PM_2.5_ on buccal MN frequency in a sub-group of 80 firefighters.

Variables	β	t	*p*-Value	95% CI
Inhaled dose (µg kg^−1^)				
PM_10_	−0.01	−0.04	0.97	−0.08 to 0.78
PM_2.5_	0.07	0.29	0.77	−0.42 to 0.57

95% CI= confident interval; β = regression coefficient.

## Data Availability

The authors do not have permission to share data.

## Supplementary Materials

The following supporting information can be downloaded at: https://www.mdpi.com/article/10.3390/toxics12030201/s1, Table S1: Summary of parameters considered for inhalation dose estimation, specifically the concentration of PM both indoor and outdoor in the seven sampled fire stations (A); the time of exposure both indoor and outdoor (B); and the inhalation rate in males and in females, by age category, unadjusted for body weight (C).

## Abbreviations

PM: Particulate matter; BMCyt: Buccal micronucleus cytome assay; DNA: Deoxyribonucleic acid; IARC: International Agency for Research on Cancer; MN: Micronucleus; BN: Binucleated cells; NBUDs: Nuclear buds; BMI: Body mass index; PPE: Personal protective equipment; NaCl: Sodium chloride; NaOH: Sodium hydroxide; EDTA: ethylenediaminetetraacetic acid tetra; HCL: hydrochloric acid; Tris-HCL: Tris-hydrochloride.
