# Supplementary material for: Firefighters’ Occupational Exposure in Preparation for Wildfire Season: Addressing Biological Impact
Source: Toxics. 2024 Mar 5;12(3):201. doi: 10.3390/toxics12030201 (PMC10974005; doi:10.3390/toxics12030201)
Supplement: Supplementary file 1 [file toxics-12-00201-s001.zip › toxics-2884264-supplementary.pdf]

Supplementary Material

Table S1. Summary of parameters considered for inhalation dose estimation, specifically the concentration of PM both indoor and outdoor in the seven sampled fire station (A); the time of exposure both indoor and outdoor (B) and the Inhalation Rate in males and in females, by age category, unadjusted for body weight (C).

Parameters considered for inhalation dose estimation:

A. Mean concentration of PM (µg/m³):

		**FS1**	**FS2**	**FS3**	**FS4**	**FS5**	**FS6**	**FS7**
**Indoors**								
	PM_{10}	16.10	7.90	7.50	11.90	9.30	11.50	9.20
	$PM_{2.5}$	14.10	6.30	6.40	10.50	7.70	9.20	6.70
**Outdoors**								
	PM_{10}	19.00	15.80	27.00	25.00	17.00	23.00	21.60
	$PM_{2.5}$	4.00	2.30	7.70	6.00	6.00	3.00	2.70

B. Time of exposure:

**Daily time spent in FC (hours/day)[a]**	**Daily time spent indoors (hours/day)[b]**	**Daily time spent indoors (min/day)**	**Daily time spent outdoors (hours/day)[c]**	**Daily time spent outdoors (min/day)**
12.00	3.60	216	8.40	504
8.00	2.40	144	5.60	336

C. Inhalation Rate by Age Group:

	**Males**		**Females**	
**Age (years)**	**Daily Inhalation Rate (m³/day)[d]**	**Inhalation Rate Min (m³/min)**	**Daily Inhalation Rate (m³/day)[e]**	**Inhalation Rate Min (m³/min)**
16 to <21	17.21	1.19×10^{-2}	13.59	9.44×10^{-3}
21 to <31	18.82	1.31×10^{-2}	14.57	1.01×10^{-2}
31 to <41	20.29	1.41×10^{-2}	14.98	1.04×10^{-2}
41 to <51	20.94	1.45×10^{-2}	16.20	1.13×10^{-2}
51 to <61	20.91	1.45×10^{-2}	16.19	1.12×10^{-2}
61 to <71	17.94	1.25×10^{-2}	12.99	9.02×10^{-3}

[a] Information retrieved from the questionnaire; [b] Considering a reported permanence of 30%; [c] Considering a reported permanence of 70%; [d] Adapted from Table 6.14 of Exposure Factors Handbook (USEPA, 2011); [e] Adapted from Table 6.15 from Exposure Factor Handbook (USEPA, 2011).
